# Utility of warning signs in guiding admission and predicting severe disease in adult dengue

**DOI:** 10.1186/1471-2334-13-498

**Published:** 2013-10-24

**Authors:** Yee-Sin Leo, Victor C Gan, Ee-Ling Ng, Ying Hao, Lee-Ching Ng, Kwoon-Yong Pok, Frederico Dimatatac, Chi-Jong Go, David C Lye

**Affiliations:** 1 Department of Infectious Disease, Communicable Disease Center, Institute of Infectious Disease and Epidemiology, Tan Tock Seng Hospital, Singapore, Singapore; 2 Saw Swee Hock School of Public Health, National University of Singapore, Singapore, Singapore; 3 Yong Loo Lin School of Medicine, National University of Singapore, Singapore, Singapore; 4 Environmental Health Institute, National Environment Agency, Singapore; School of Biological Science, Nanyang Technological University, Singapore, Singapore

**Keywords:** Dengue, Warning signs, Utility, Disease progression, Admission

## Abstract

**Background:**

The recommendation from the 2009 World Health Organization guidelines for managing dengue suggests that patients with any warning sign can be hospitalized for observation and management. We evaluated the utility of using warning signs to guide hospital admission and predict disease progression in adults.

**Methods:**

We conducted a prospective cohort study from January 2010 to September 2012. Daily demographic, clinical and laboratory data were collected from adult dengue patients. Warning signs were recorded. The proportion of admitted patients using current admission criteria and warning signs was compared. The sensitivity, specificity, positive and negative predictive values of warning signs in predicting disease progression were also evaluated.

**Results:**

Four hundred and ninety-nine patients with confirmed dengue were analyzed. Using warning signs instead of the current admission criteria will lead to a 44% and 31% increase in admission for DHF II-IV and SD cases respectively. The proportion of non-severe dengue cases which were admitted also increased by 32% for non DHF II-IV and 33% for non-SD cases. Absence of any warning signs had a NPV of 91%, 100% and 100% for DHF I-IV, DHF II-IV and SD. Of those who progressed to severe illness, 16.3% had warning signs on the same day while 51.3% had warning signs the day before developing severe illness, respectively.

**Conclusions:**

Our findings demonstrated that patients without any warning signs can be managed safely with ambulatory care to reduce hospital resource burden. No single warning sign can independently predict disease progression. The window from onset of warning sign to severe illness in most cases was within one day.

## Background

Dengue is the most common arboviral infection. It has a wide disease spectrum, ranging from a self-limiting fever to severe disease with fatal outcome. The World Health Organization (WHO) estimates that 2.5 billion people are at risk of dengue. A recent model estimates 390 million infections annually with 96 million cases of clinically apparent disease [1]. More than half a million people develop severe illness, i.e., dengue hemorrhagic fever (DHF) or dengue shock syndrome (DSS) or severe dengue (SD). The case fatality rate reported for DHF from 2000–2007 in the Americas was 1.2% [2].

The WHO published two sets of guidelines for managing dengue: the 1975–1997 guidelines and the recent 2009 guidelines. In the 1975–1997 guidelines, dengue severity was categorized into dengue fever (DF), DHF and DSS [3]. To be classified as DHF, all four of the following requirements: fever, hemorrhagic manifestations, thrombocytopenia and plasma leakage must be met. This classification was, however, not inclusive enough for all forms of severe outcomes [4]. The 2009 WHO guidelines categorized disease severity into dengue without warning sign(s) (WS), dengue with WS and SD [2].

The 2009 WHO guidelines proposed using WS as indicators for disease progression. The WS comprised abdominal pain, persistent vomiting, clinical fluid accumulation, mucosal bleed, lethargy, hepatomegaly (liver enlargement by >2 cm) and an increase in hematocrit, concurrent with a rapid decrease in platelet count [2]. The WHO recommended patients with any WS be hospitalized for observation and management.

Singapore, an economically well-developed city in South-east Asia, has predominantly adult dengue cases [5,6]. We assessed the utility of WS in guiding hospital admission and evaluated its predictive value for disease progression among adults. In a retrospective study, we previously reported that persistent vomiting, hepatomegaly, clinical fluid accumulation, hematocrit rise together with platelet decrease were highly specific for predicting DHF and SD; however none of the WS were sensitive [7]. Notably, in the absence of any WS, the negative predictive value was 96% and 97% for DHF and SD respectively, suggesting that disease progression was extremely unlikely in the absence of any WS. We validated the findings in this prospective study aiming to assess the utility of WS in guiding hospital admission and predicting disease progression to DHF and SD.

## Methods

### Patients

We enrolled adult patients who consented to take part in the Prospective Adult Dengue Study, a cohort study of acutely febrile adults at the Communicable Disease Center (CDC), Tan Tock Seng Hospital, Singapore from January 2010 to September 2012. These comprised referrals for fever for investigation from the emergency department (ED), other medical institutions, or self-referral to the CDC. Inclusion criteria were age 18 years and above with acute undifferentiated febrile illness (recorded temperature >37•5°C with no alternative clinical diagnosis). Pregnant women were excluded from the study.

This study compared the baseline characteristics and outcomes of two cohorts; those admitted from ED who were enrolled as inpatients (ED cohort) and those initially enrolled in outpatient setting at the research clinic (outpatient cohort). The outpatient cohort was the basis to evaluate the utility of WS in guiding admission. The entire cohort was analyzed to assess the predictive value of WS for disease progression. All outpatients were managed at the Infectious Disease Research Clinic at CDC. Outpatients were managed by three trained Medical Officers on a daily basis during acute illness until initiation of the recovery phase and reviewed at 21–30 days after study enrolment. Decision to admit patients from both the research clinic and ED were based on the published hospital admission criteria [8] and the attending physician’s judgment. Criteria for recommending admission include: platelet count ≤50 000/mm^3^, serum hematocrit ≥50%, systolic blood pressure ≤90 mmHg, postural drop in blood pressure >20 mmHg, pulse ≥100/min, clinical bleeding (except petechiae), patients with severe abdominal pain and persistent vomiting, elderly patients with comorbidities, and whether patients fulfilled our DHF predictive model [9,10].

To evaluate the utility of WS in guiding admission, we compared admission based on published admission criteria and Medical Officers’ judgment with alternative hypothetical scenario of WS-guided admission. The clinical outcomes were categorized into three groups (i) DHF I-IV, (ii) DHF II-IV as defined by the WHO 1997 guidelines [3] and (iii) SD as defined in WHO 2009 guidelines [2].

Dengue viral infection was confirmed by RT-PCR [11] or NS1 detection by Dengue NS1 Ag Strip (Bio-Rad Laboratories, Marnes-la-Coquette, France) at the Environment Health Institute, Singapore, a WHO Collaborating Center for Reference and Research on Arbovirus and their Associated Vectors. Detailed demographic, clinical, and laboratory data were prospectively collected according to research protocols. Warning signs were also recorded.

### Warning signs

Warning signs assessed comprised abdominal pain (or tenderness), persistent vomiting, mucosal bleeding, clinical fluid accumulation, hepatomegaly (>2 cm) and increase in hematocrit (with concurrent decrease in platelet) [2]. We opted to exclude lethargy as almost all patients reported subjective lethargy. Lethargy as a WS was also removed in our previous analysis due to ambiguity in patients’ interpretation of lethargy and lack of objective differentiation from tiredness [7]. The sensitivity (Sn), specificity (Sp), positive predictive value (PPV) and negative predictive value (NPV) of each WS for predicting disease progression was evaluated.

### Clinical outcomes

We utilized both sets of the WHO guidelines in defining disease severity. DHF was defined as per WHO 1997 guidelines, namely concomitant high fever, thrombocytopenia, hemorrhagic manifestations and plasma leakage [3]. Plasma leakage was determined by physical examination or chest radiography showing fluid accumulation or hematocrit change of ≥20% or hypoproteinemia. DHF grade I was defined as presence of mild bleeding phenomena such as petechiae, DHF II additionally required the occurrence of spontaneous bleeding such as mucosal or gastrointestinal bleeding. All DHF III and IV cases were classified as DSS. For DSS, DHF cases required either (i) tachycardia (pulse >100/minute) with narrow pulse pressure (<20 mmHg) or (ii) systolic blood pressure (SBP) <90 mmHg [12].

SD was defined as per WHO 2009 guidelines namely cases with severe plasma leakage (shock, fluid accumulation with respiratory distress), severe bleeding or severe organ impairment [2].

### Statistical analysis

Categorical variables were described as absolute numbers and percentages. Chi-square tests were used to compare categorical variables between groups of different clinical outcomes. Median values and percentile ranges were used in descriptive analysis for continuous variables. Wilcoxon or t-tests were used for hypothesis testing.

To analyze proportion of cases admitted when comparing admission based on published criteria with WS-guided admission, patients were classified into three categories of severe illness; (i) DHF I-IV, (ii) DHF II-IV or (iii) SD. Proportions of admitted severe and non-severe cases were compared between admission practices (current practice versus (vs) WS-guided). For predicting clinical outcomes of DHF I-IV, DHF II-IV and SD, patients with presence of WS and severe dengue were classified as true positives (TP). When assessing such prediction of clinical outcome, cases already with severe outcome on enrolment were excluded from analysis. Patients with WS but who did not progress to severe dengue were classified as false positives (FP). Sensitivity (Sn), specificity (Sp), positive predictive value (PPV) and negative predictive value (NPV) were calculated using WS to predict the clinical outcomes of DHF I-IV, DHF II-IV and SD. The performance of using individual WS and combinations of two and three WS were tested. All statistical analyses were performed in the R statistical environment and conducted at the significance level of 0.05.

### Ethics approval

Ethical approval was provided by the Domain Specific Review Board of the National Healthcare Group, Singapore (DSRB/E/2009/432). Written informed consent was obtained from all subjects.

## Results

### Demographics

Four hundred and ninety-nine patients with confirmed dengue were enrolled and analyzed in this study. Three hundred and seventy-six patients were recruited from the research clinic and were described as the “outpatient” cohort. The remaining 123 patients were recruited after admission from ED and described as the “ED” cohort. Of the outpatient cohort, 77 patients were subsequently admitted while 299 patients continued their treatment as outpatients.

The demographics between the two cohorts were similar except a small difference in the median age of the ED cohort. There were significantly more DHF patients (p < 0.001) in ED cohort (Table 1).

**Table 1:** Demographic comparison of ED and outpatient cohorts

	**ED**	**Outpatients**	**P-value**
	**N = 123**	**N = 376**	
**Male**	92 (74.8)	304 (81.0)	0.19
**Age (years)**	37 (22, 58)	33 (21, 47)	0.02
**Illness day on recruitment**	6 (3, 8)	6 (3, 8)	0.17
**Warning signs**	69 (56.1)	203 (54.0)	0.76
**DHF**	68 (55.3)	70 (18.6)	<0.001

For categorical variables, absolute numbers (and the relative percentage) are being indicated. For continuous variables, medians (and the relative 5^th^-95^th^ percentile) are being indicated. DHF = dengue hemorrhagic fever.

In the ED cohort, 69 (56.1%) had at least one WS. The frequencies of WS did not differ significantly between gender (p = 0.64) and age (p = 0.88). Patients were unwell for a median of 6 days (5^th^ – 95^th^ percentile; 3 – 8 days) at the time of enrolment and this did not differ significantly between patients with and without WS (p = 0.36) (Table 2).

**Table 2:** Comparison of demographic data and outcomes in ED and outpatient cohorts with and without warning signs

	**ED (N = 123)**			**Outpatients (N = 376)**		
	**WS + ve (N = 69)**	**WS -ve (N = 54)**	**P-value**	**WS + ve (N = 203)**	**WS -ve (N = 173)**	**P-value**
**Male**	50 (72.5)	42 (77.8)	0.64	162 (79.8)	142 (82)	0.67
**Age (years)**	36 (23, 53)	37 (21, 60)	0.88	34 (22, 47)	32 (32, 48)	0.68
**Illness day on recruitment**	6 (3, 8)	6 (3, 8)	0.36	6 (3, 8)	6 (4, 9)	0.06
**DHF I-IV**	45 (65.2)	23 (42.6)	0.02	55 (27.0)	15 (8.7)	<0.001
**DSS (DHF III, IV)**	6 (8.1)	1 (1.9)	NA	6 (3.0)	0	NA
**SD**	7 (10.1)	0	NA	13 (6.4)	0	NA
**Admission**	NA		NA	52 (25.5)	25 (14.5)	0.01

For categorical variables, absolute numbers (and the relative percentage) are being indicated. For continuous variables, medians (and the relative 5^th^-95^th^ percentile) are being indicated.

*WS* Warning signs, *DHF* dengue hemorrhagic fever, *DSS* dengue shock syndrome, *SD* severe dengue.

In the outpatient cohort of 376 cases, 203 (54%) had at least one WS, 77 (20.5%) were admitted and 70 (18.6%) fulfilled DHF criteria. Of these 70 DHF cases, 6 cases were classified as DSS. Independently, 13 would be classified as SD using WHO 2009 classification (Table 2). Three DSS patients were not admitted. Two had single readings of transient hypotension (systolic blood pressure less than 90 mmHg) while the third had persistent low baseline blood pressure. All three remained clinically stable throughout the entire course of illness. Within the same cohort, there were another 4 SD patients who were not admitted. Three reported menorrhagia without the need for transfusion and one had isolated raised transaminases with AST 1040 U/L and ALT 1029 U/L. Their conditions improved during outpatient care and all four were also clinically stable throughout the entire course of illness. There was a single mortality, previously published as a case report [13]. He was admitted because of loss of appetite, severe vomiting, nausea, malaise, mild headaches and dizziness. He was alert and hemodynamically stable at the time of admission but became restless in the ward and died abruptly less than twelve hours after admission. He did not fulfill DHF criteria. Post-mortem investigation showed evidence of myocarditis.

Similarly, in the outpatient cohort, the frequencies of WS did not differ significantly between gender (p = 0.67) and age (p = 0.68). Most patients had illness onset of 6 days (5^th^ – 95^th^ percentile; 3 – 8 days) at enrolment; this did not differ significantly between the group of patients with and without WS (p = 0.06) (Table 2).

In both cohorts, WS were present in significantly higher proportion of cases with DHF. All patients with severe illness defined as DSS and SD had at least one WS except for one DSS patient. This patient was classified as DSS with a single episode of hypotension (SBP <90 mmHg) and evidence of plasma leakage comprising hypoproteinemia without >20% hematocrit change [12]. The statistical significance of the observations was not tested in the SD and DSS patients in both cohorts as the number of patients in these categories were too small. Of note, a significantly higher proportion of those with WS were admitted (p =0.01) (Table 2).

### Hospital admission criteria and Medical Officers’ judgment vs WS-guided admission

Admission based on the approach of using the hospital admission criteria and Medical Officers’ judgment was compared with the hypothetical scenario of WS-guided admission (Table 3). Although WS-guided admission included all severe dengue case (DHF II-IV and SD), WS-guided admission would lead to an increase in proportion admitted of 44% and 31% for DHF II-IV and SD, respectively. The proportion of non-severe dengue patients who was admitted also increased by 32% for non DHF II-IV and 33% for non-SD patients. The increase in admissions was significant in all categories of dengue patients, except for SD patients.

**Table 3:** Proportion of severe vs non-severe dengue cases admitted from ambulatory care using either current admission practice or using warning signs as admission criteria (percentage of cases (95% confidence interval))

	**DHF I-IV**	**P-value**	**Non-DHF I-IV**	**P-value**
**Admission criteria + clinical judgment**	59% (46%, 70%)	0.02	12% (8%, 16%)	<0.01
**WS**	79% (67%, 87%)		48% (43%, 54%)	
	**DHF II-IV**	**P-value**	**Non-DHF II-IV**	**P-value**
**Admission criteria + clinical judgment**	56% (4%, 71%)	<0.01	16% (12%, 20%)	<0.01
**WS**	100.0% (88%, 100%)		48% (43%, 54%)	
	**SD**	**P-value**	**Non-SD**	**P-value**
**Admission criteria + clinical judgment**	69% (39%, 91%)	0.13	19% (15%, 23%)	<0.01
**WS**	100.0% (66%, 100%)		52% (47%, 58%)	

*WS* Warning signs, *DHF* dengue hemorrhagic fever, *SD* severe dengue.

P-values indicate the significance of the difference in proportion between current admission practice and using warning signs as admission criteria.

### WS in predicting disease progression

Mucosal bleeding and abdominal pain were the two most common WS at 75.9% and 43.3%, respectively. In analyzing the performance of individual WS in predicting DHF and SD, we found mucosal bleeding had the highest sensitivity (DHF I-IV: 61%; DHF II-IV: 100%; SD: 62%). The NPV for mucosal bleeding was 100% for DHF II-IV and 98% for SD. However, both specificity and PPV for prediction of DHF and SD were relatively low (Table 4). Having any of the WS would have 100% sensitivity in predicting DHF II-IV and SD. However, the specificity was only 52% and 48% for DHF II-IV and SD, respectively. The PPV of any WS was low at 21% and 6% for DHF II-IV and SD, respectively. Notably, the absence of any WS had NPV of 91%, 100% and 100% for DHF I-IV, DHF II-IV and SD, respectively. Having two or three of the WS, however, decreased the overall sensitivity and NPV in predicting DHF I-IV, DHF II-IV and SD but the specificity and PPV in predicting disease progression were increased in all categories of disease (Table 4).

**Table 4:** Performance of individual warning signs in predicting DHF and SD in outpatients

**Warning sign**	**DHF I-IV (N = 70)**				**DHF II-IV (N = 43)**				**SD (N = 13)**			
	**Sn**	**Sp**	**PPV**	**NPV**	**Sn**	**Sp**	**PPV**	**NPV**	**Sn**	**Sp**	**PPV**	**NPV**
Abdominal pain (N = 88)	31	78	25	83	37	78	18	91	38	77	6	97
Persistent vomiting (N = 16)	7	96	31	82	9	96	25	89	23	96	19	97
Clinical fluid accumulation (N = 1)	1	100	100	82	0	100	0	89	0	100	0	97
Mucosal bleeding (N = 154)	61	64	28	88	100	67	28	100	62	60	5	98
Hepatomegaly (> 2 cm) (N = 2)	1	100	50	82	0	99	0	89	0	99	0	97
↑ in hematocrit; rapid ↓ of platelet (N = 10)	14	100	100	84	9	98	40	89	31	98	40	98
Any warning sign (N = 203)	79	52	27	91	100	52	21	100	100	48	6	100
Two warning signs (N = 61)	33	88	38	85	47	88	33	93	46	85	10	98
Three warning signs (N = 7)	6	99	57	82	9	99	57	89	8	98	14	97

Numbers indicated are percentages.

*Sn* sensitivity, *Sp* specificity, *PPV* Positive predictive value, *NPV* Negative predictive value.

Combinations of two warning signs include: Abdominal pain and mucosal bleeding; Abdominal pain and persistent vomiting; Abdominal pain and hepatomegaly; Abdominal pain and clinical fluid accumulation; Mucosal bleeding and persistent vomiting; Mucosal bleeding and hepatomegaly; Mucosal bleeding and change in hematocrit level; Abdominal pain and change in hematocrit level.

Combinations of three warning signs include: Abdominal pain, mucosal bleeding and persistent vomiting; Abdominal pain, mucosal bleeding and change in hematocrit level.

*WS* Warning signs, *DHF* dengue hemorrhagic fever, *SD* severe dengue.

**Figure 1:**
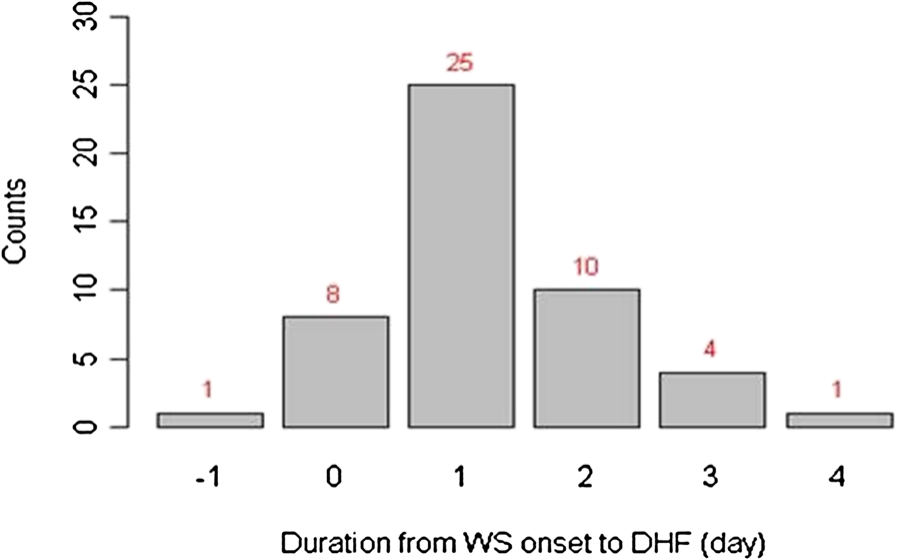
Duration from onset of warning signs to DHF in outpatients (N = 49).

We further identified 49 cases in the outpatient cohort who progressed to severe illness: 8 (16.3%) had WS the same day as they progressed to severe illness and 25 (51%) cases had WS one day before progressing to severe illness (Figure 1).

We further analyzed mucosal bleeding as it was the most frequently reported WS. Eighty-four percent and 68% of DF and DHF patients, respectively, had gingival bleeding. Epistaxis was the next most frequent mucosal bleeding event, followed by menorrhagia in both DF and DHF patients. However, there was no significant difference in the frequency of these mucosal bleeding events (namely gingival bleeding, epistaxis, hemoptysis or hematuria) between DF and DHF patients (data not shown).

## Discussion

The revised classification was well-received in a multi-center survey conducted from February to November 2009. However, participants of the study also indicated that the revised classification may result in over-admission since the WS in the WHO 2009 guidelines was less specific for the diagnosis of probable dengue [14]. Several other studies compared sensitivity of the two sets of the WHO guidelines in classifying severe illness [15-18]. Three of these studies indicated that the new classification was highly sensitive in identifying severe illness. In a recently published study conducted in Lucknow, India, the new set of guidelines identified 98% of all severe illness [18]. Narvaez *et al.*[17] reported in a separate study that the revised classification was highly sensitive in identifying cases which need heightened care; however it was noted that it was less specific and can no longer differentiate the pathogenic entity of DHF and DSS. In addition, they noted that DENV2 was significantly associated with DHF/DSS but not with the revised classification for severe illness. Similar findings were reported in other pediatric studies [19,20].

Kalayanarooj *et al.*, also noted that WHO 2009 classification may have been too sensitive and would have created about 2 times additional workload to medical personnel [15]. This is undesirable particularly in resource-limited endemic regions. Our study suggested a 2–3 fold increase in workload if the hospital adopted WS-guided admission over the current hospital admission criteria and clincian’s judgment.

The WHO recommended that dengue patient with any WS be admitted for close observation [2]. In Singapore, dengue incidence averages 5000 cases annually since 2010 [21-23]. The admission rate to public hospitals was approximately 37% of all cases notified to Ministry of Health in 2012 (unpublished data). This was a drop from approximately 80% admission rate in 2000–2004 [8]. The drop in admission may be due to the establishment of a national guideline for management of dengue patients in 2005 [24]. In this study, we observed that 54% of the outpatient cohort had WS. In contrast, using the hospital admission criteria and clinical judgment, only 25.6% of outpatients with WS were admitted. Patients with WS who were managed as outpatients did not have any adverse outcomes did just as well. Although some cases with WS who were classified as having severe illness were not admitted, none had adverse outcomes. This could be argued as under-hospitalization and this practice cannot be generalizable to healthcare settings lacking dedicated dengue care units that can closely monitor the cases. We found that with trained staff reviewing cases daily, subjects with isolated elevation of transaminases or a single brief hypotensive episode can be safely managed in the outpatient setting. The increased resources needed for the higher hospitalization rates if WS were used as admission criteria have to be balanced against the careful individualized management of cases in ambulatory care using clinical judgment to determine admission.

Although the presence of any WS was strongly associated with severe outcomes, it lacked specificity and had a low PPV. We previously reported in a large retrospective study that absence of any WS gave a high NPV for severe illness progression [7]. The findings of this prospective study supported our previous results. In the absence of any WS, NPV was 91%, 100% and 100% for DHF I-IV, DHF II-IV and SD. These data supported the proposal of using WS to screen those who do not need hospital admission. Tsai *et al.*, had also previously supported the proposal of outpatient treatment for dengue patients who do not have any WS [16]. For patients with WS, we demonstrated that with close monitoring it was safe to continue outpatient management in majority of the cases in our center. Nonetheless, the decision to admit patients with WS would still be dependent on access to healthcare facilities and local practice.

It is critical to understand the temporal onset of WS to severe illness and the frequencies of individual WS in severe illness [7,25]. In a multi-center study, Alexander *et al.*, had noted that in the majority of patients who developed severe illness, WS developed 1 day prior to the requirement for intervention and 4–7 days since development of illness [25]. They observed that abdominal pain, lethargy, decreased platelet count and mucosal bleeding were strongly associated with development of severe illness. At least one of these WS was also found to be associated with severe illness in several other studies [7,16,26]. We found that WS typically occurred one day prior to the development of severe illness. The window to provide intervention can be challenging in areas where healthcare facilities are remote from place of residence. We reported a single fatality that had WS one day before his death, highlighting the possible rapid progression of dengue disease [13]. With regard to mucosal bleeding as the most frequent WS, its frequency was not statistically different between the DF and DHF patients. Individual bleeding symptoms were also not statistically different between DF and DHF patients. Our observation would support the lack of evidence for a significantly higher rate of hemorrhage in DHF patients.

The limitations of this study include the tertiary setting of the hospital that does not allow the results from this study to be generalized to primary healthcare settings where medical expertise and resources are less readily available, the small sample size which prevented detailed analysis on the performance of each individual WS and the adult cohort that cannot be generalized to the pediatric population. Because DENV-2 was the predominant serotype during the study period and our cohort had low prevalence of co-morbidities, serotypes and presence of co-morbidities in relation to WS and disease outcomes were not evaluated.

## Conclusions

This prospective study demonstrated a strong correlation between absence of WS and disease non-progression. The absence of WS can therefore be utilized as an indicator to safely manage cases in outpatient settings. However, none of the individual WS can independently predict disease progression but having two or more WS warrants close observation. Further work in identifying other laboratory and/or clinical markers is needed to improve dengue management.

## Competing interests

Yee-Sin Leo was a medical advisor to Sanofi-Pasteur for a dengue vaccine trial. The authors declare that they have no competing interests.

## Authors’ contributions

*Concept, design, data acquisition, data analysis and data interpretation.* YSL: Concept design and data interpretation; VCG, LCN, KYP, DF and CJG: Data acquisition; ELN: Data interpretation; YH: Data analysis; DCL: Concept design. *Drafting or revising manuscript.* YSL, VCG ELN: Drafting and revising; YH, LCN, KYP, DF, CJG, DCL: Revising. All authors read and approved the final manuscript.

## Pre-publication history

The pre-publication history for this paper can be accessed here:

http://www.biomedcentral.com/1471-2334/13/498/prepub
